# Mechanical Aortic Valve Thrombosis: Treatment With Tissue Plasminogen Activator

**DOI:** 10.1016/j.jaccas.2025.104006

**Published:** 2025-07-16

**Authors:** Ghazala Munawar, Vasu Gupta, Zenab Laiq, Khaled Sleik

**Affiliations:** a Department of Internal Medicine, Northwest School of Medicine, Peshawar, Pakistan; b Department of Internal Medicine, Cleveland Clinic Akron General, Akron, Ohio, USA; c Department of Cardiovascular Medicine, Heart, Vascular and Thoracic Institute, Cleveland Clinic Akron General, Akron, Ohio, USA

**Keywords:** mechanical aortic valve, mechanical aortic valve thrombosis, tissue plasminogen activator

## Abstract

**Background:**

Mechanical aortic valve thrombosis is a rare complication in patients with mechanical valve replacements. Despite the advantages of mechanical valves, they necessitate lifelong anticoagulation therapy to prevent thrombotic events.

**Case Summary:**

A 32-year-old man with a mechanical aortic valve presented with chest pain and dyspnea suggestive of myocardial infarction. Left heart catheterization revealed 100% occlusion of the distal left circumflex artery whereas cardiac computed tomography revealed a thrombus on the mechanical aortic valve. The patient was successfully treated with thrombolytic therapy using slow intravenous Alteplase infusion.

## Graphical Abstract

**Figure:**
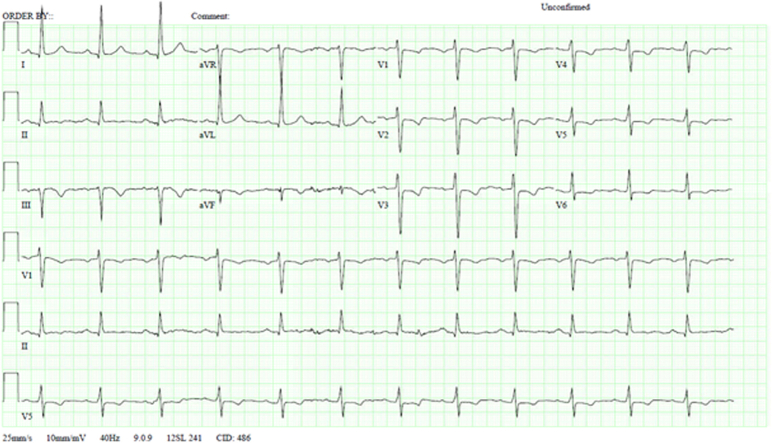

Thrombosis of bioprosthetic valves commonly occurs in the first 3 months after implantation with an annual incidence rate of 0.1% to 5.7%.^1^ It is less common in bioprosthetic valves as compared with mechanical valves and tends to affect mitral and tricuspid valves more than the aortic valve. The numbers might underestimate the annual rate because the current guidelines recommend obtaining transthoracic echocardiography (TTE) or transesophageal echocardiography (TEE) imaging only if clinically indicated and not routinely.^2^ In patients with suspected acute mechanical valve thrombosis, there is Class I recommendation to obtain urgent TTE, TEE, fluoroscopy, and/or multidetector cardiac computed tomography (CT) to assess valve function.^2^ A multidetector cardiac CT might be useful if the results of TTE, TEE, or fluoroscopy are inconclusive and can also help differentiate between pannus and thrombus. A cutoff of ≥145 Hounsfield units (HU) likely represents a pannus, with values below this more likely representing a thrombus.^3^

**Take-Home Messages**

• The case highlights the importance of multimodality imaging including TTE, TEE, cinefluoroscopy, and cardiac CT in diagnosing MAVT.
• Ultra slow infusion of tPA is a safe alternative to surgery for treatment of MAVT.

Anticoagulation with a vitamin K antagonist is recommended in patients with a mechanical prosthetic valve. The goal international normalized ratio (INR) is 2.5 for patients with aortic valve replacement without any risk factors for thromboembolism, and an INR of 3.0 is recommended in those at risk for thromboembolism (atrial fibrillation, prior thromboembolism, or hypercoagulable state).^2^ Mechanical valve dysfunction can present early as dehiscence, paravalvular leak, or endocarditis; or valvular dysfunction can present late as pannus, thrombosis, or thromboembolism.^4^ Acute mechanical aortic value thrombosis (MAVT) can present with a spectrum of symptoms, depending on the degree of obstruction or regurgitation. Patients usually present with decompensated heart failure, but cardiogenic shock on the initial presentation is not uncommon. As per the recent 2020 American Heart Association/American College of Cardiology guidelines, in patients presenting with symptoms of acute valve thrombosis, urgent intervention with either slow-infusion, low-dose fibrinolytic or emergency surgery is recommended.^2^

## Case Summary

A 32-year-old man presented to the emergency department with a 12-hour history of chest pain and dyspnea on exertion. The cardiovascular examination revealed a regular S1 and crisp, mechanical S2 along with a 3/6 early peaking systolic ejection murmur at the upper sternal border with radiation to the neck.

The patient had a history of congenital bicuspid aortic valve with severe aortic stenosis, and he underwent his first mechanical aortic valve repair in 2004. Unfortunately, he developed endocarditis shortly after, which necessitated a homograft aortic valve replacement within the same year. The patient remained stable for 8 years until a recurrence of endocarditis on the replaced valve in 2012, which led to another surgical intervention: a mechanical aortic valve replacement with On-X aortic valve size 25 mm (Artivion). The patient was maintained on warfarin with goal INR of 2-3.

**Figure 1:**
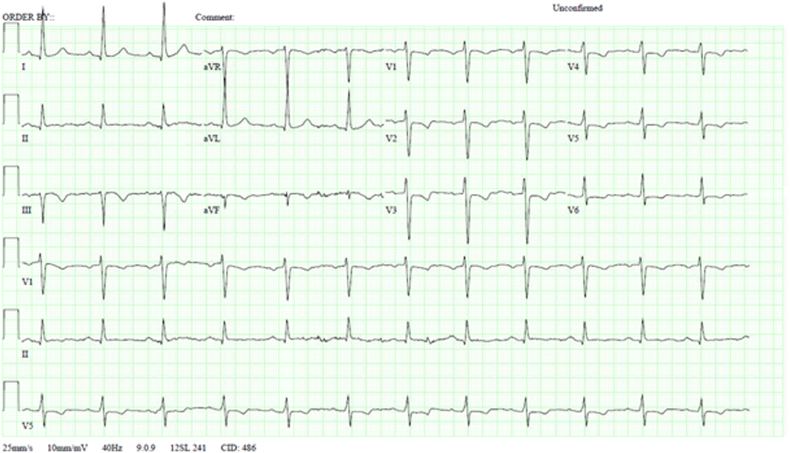
Electrocardiogram on Admission Electrocardiogram shows normal sinus rhythm with ST-segment and T-wave abnormality in anterolateral leads.

**Figure 2:**
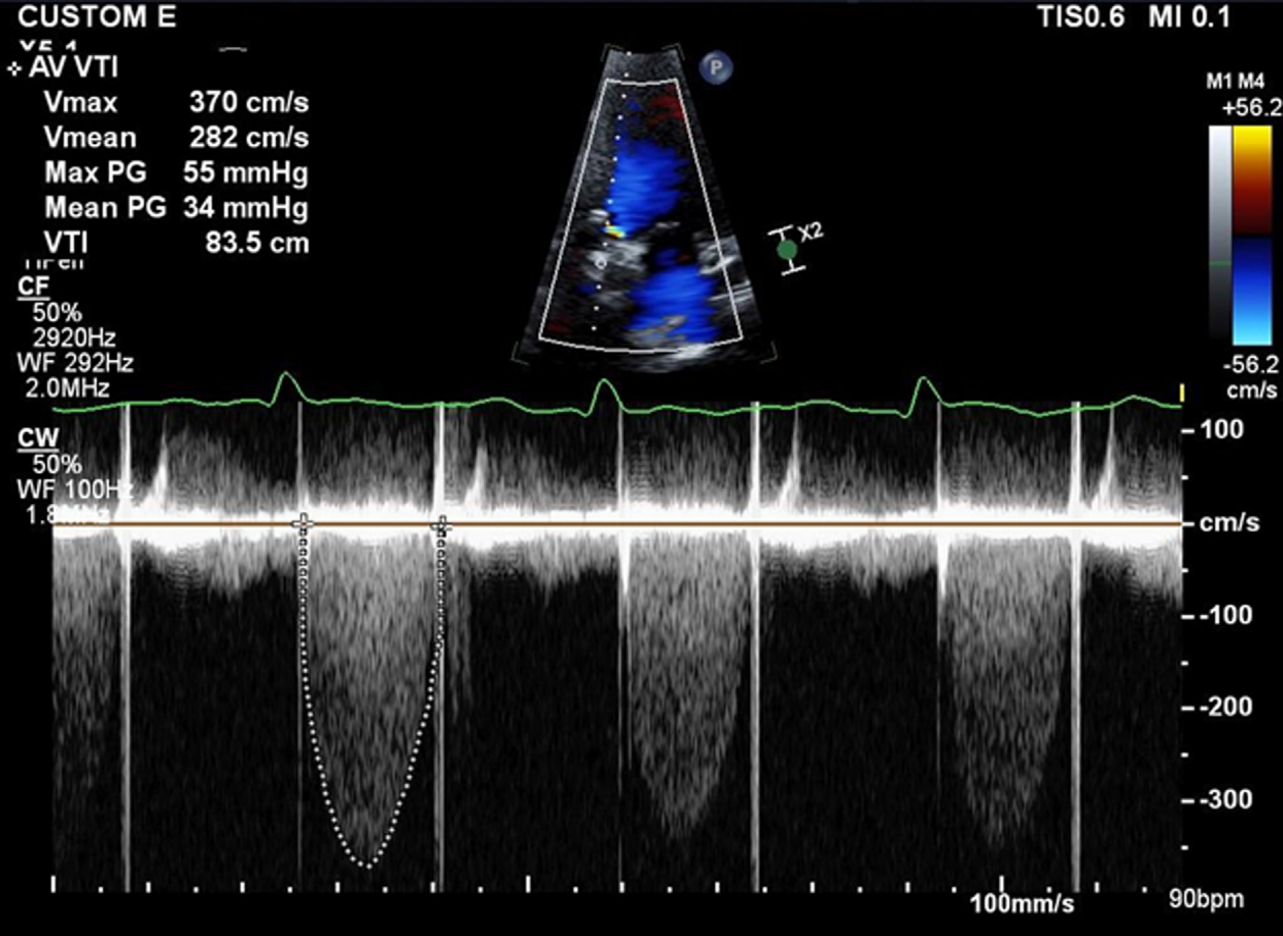
Transthoracic Echocardiography Before Tissue Plasminogen Activator Transthoracic echocardiography showing elevated peak and mean aortic transvalvular gradient of 55 mm Hg and 34 mm Hg, respectively.

His electrocardiogram showed sinus rhythm with ST-segment and T-wave abnormality in anterolateral leads, which was concerning for ischemia (Figure 1). The TTE revealed left ventricular hypertrophy with a left ventricular ejection fraction of 40% and severe hypokinesis of the inferior myocardial segments. The peak aortic valve velocity was 3.5 m/s with peak and mean gradients of 55 mm Hg and 31 mm Hg, respectively (Figure 2). The aortic valve gradients were significantly higher compared with a previous echocardiogram performed 4 years ago.

**Figure 3:**
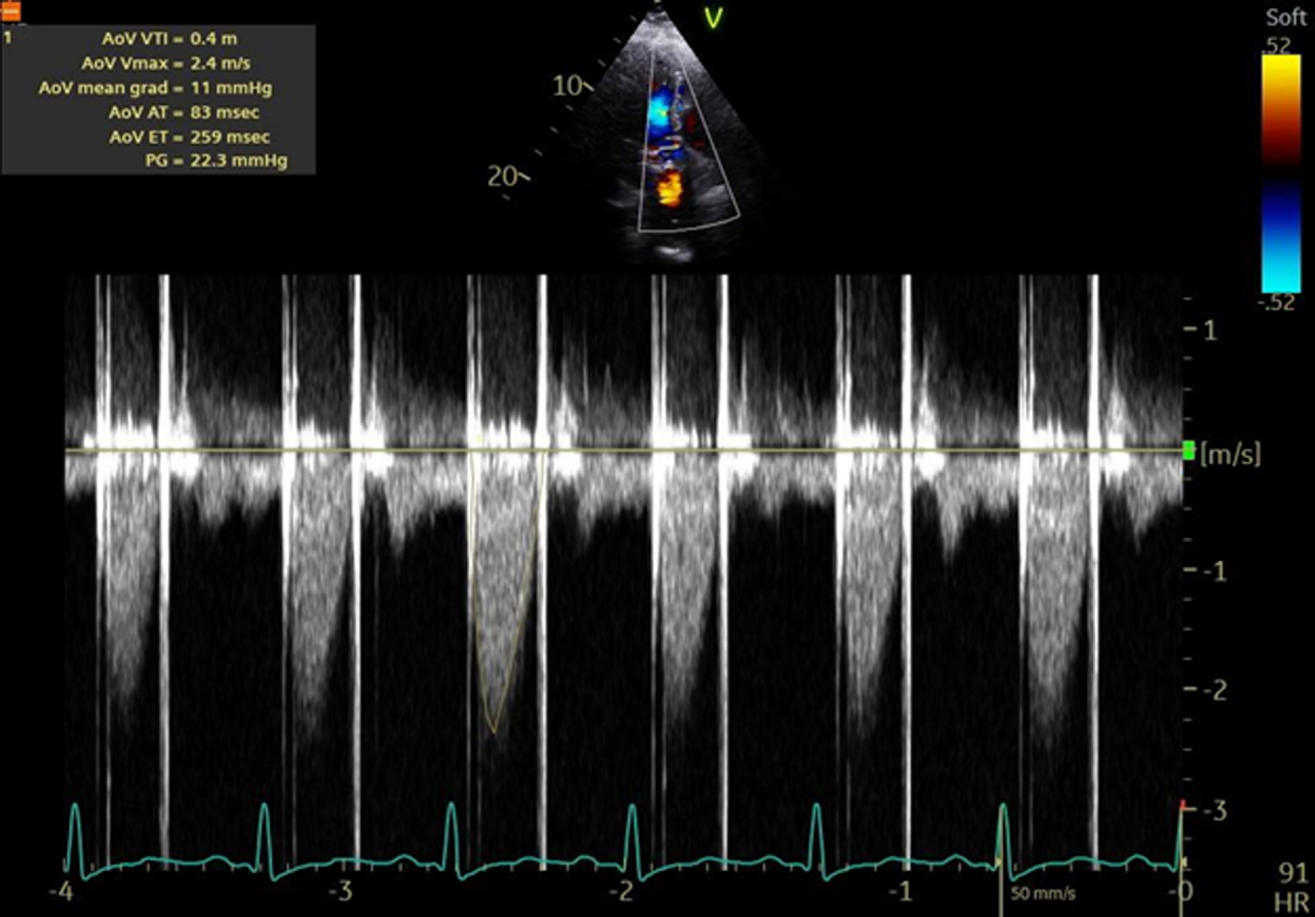
Cardiac Computed Tomography with Intravenous Contrast Cardiac computed tomography was obtained for better characterization of the valve and to rule out any thrombosis. The image shows areas of hypoattenuation on the ventricular side of the mechanical aortic valve. The mean Hounsfield units of both areas as marked by the circles (41.1 and 63.3) are much lower than the circle marking the aortic area (178) concerning for a clot/thrombus of the mechanical aortic valve.

Left heart catheterization showed 100% occlusion of a dominant left circumflex artery in the distal atrioventricular groove before the posterior descending artery branch (Video 1). The vessel was too small to be intervened. A cinefluoroscopy was performed in the cardiac catheterization laboratory and showed an immobile anterior mechanical aortic leaflet (Video 2). Cardiac computed tomography (CT) was performed for optimal assessment of the aortic valve, and it demonstrated multiple thrombi on the ventricular aspect of the mechanical aortic valve (Figure 3). Motion of the aortic valve leaflets was severely restricted, with the left leaflet being more affected than right along with restricted valve opening. Mechanical aortic valve thrombosis was diagnosed with myocardial infarction secondary to thromboembolism to the left circumflex artery.

## Management

**Figure 4:**
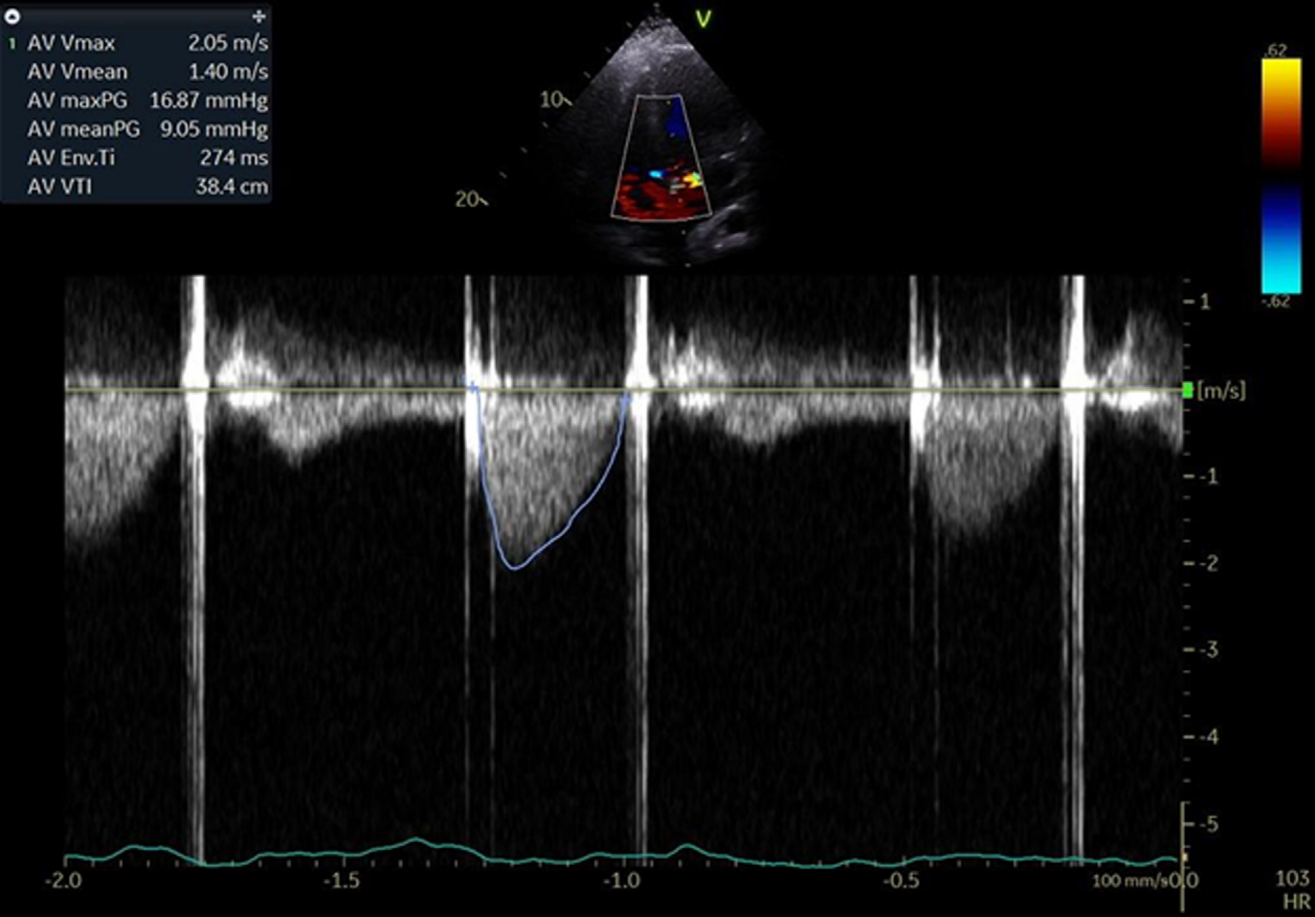
Transthoracic Echocardiography After the Second Cycle of Tissue Plasminogen Activator Transthoracic echocardiography showing a much improved peak and mean transvalvular gradient of 16 mm Hg and 9 mm Hg, respectively.

The case was particularly challenging due to the patient’s history of multiple mechanical aortic valve replacements. We considered the pros and cons of performing another mechanical valve replacement versus trying thrombolytic therapy for valve thrombosis. After deliberation, we opted for a low-dose (25 mg), ultra slow infusion (24 hours) of tissue-type tissue plasminogen activator (tPA), with the option for repetition if necessary. This protocol was based on the PROMETEE (Prosthetic Mechanical Valve Thrombosis and the Predictors of Outcome) ultra slow trial.^5^ The patient receive d a slow infusion of tPA at the rate of 0.04 mg/mL over 25 hours via a peripheral intravenous line, followed by a 6-hour infusion of weight-based therapeutic unfractionated heparin. A repeat TTE showed partial improvement with peak and mean aortic valve gradients of 27 mm Hg and 16 mm Hg, respectively, prompting a second tPA infusion the following day. We performed TTE after the second tPA infusion (50 mg over 72 hours) which revealed no obvious thrombus and reduced aortic valve gradients with a peak and mean aortic valve gradient of 17 mm Hg and 9 mm Hg, respectively (Figure 4). Cinefluoroscopy performed after the tPA infusion revealed markedly improved valve opening (Video 3).

**Figure 5:**
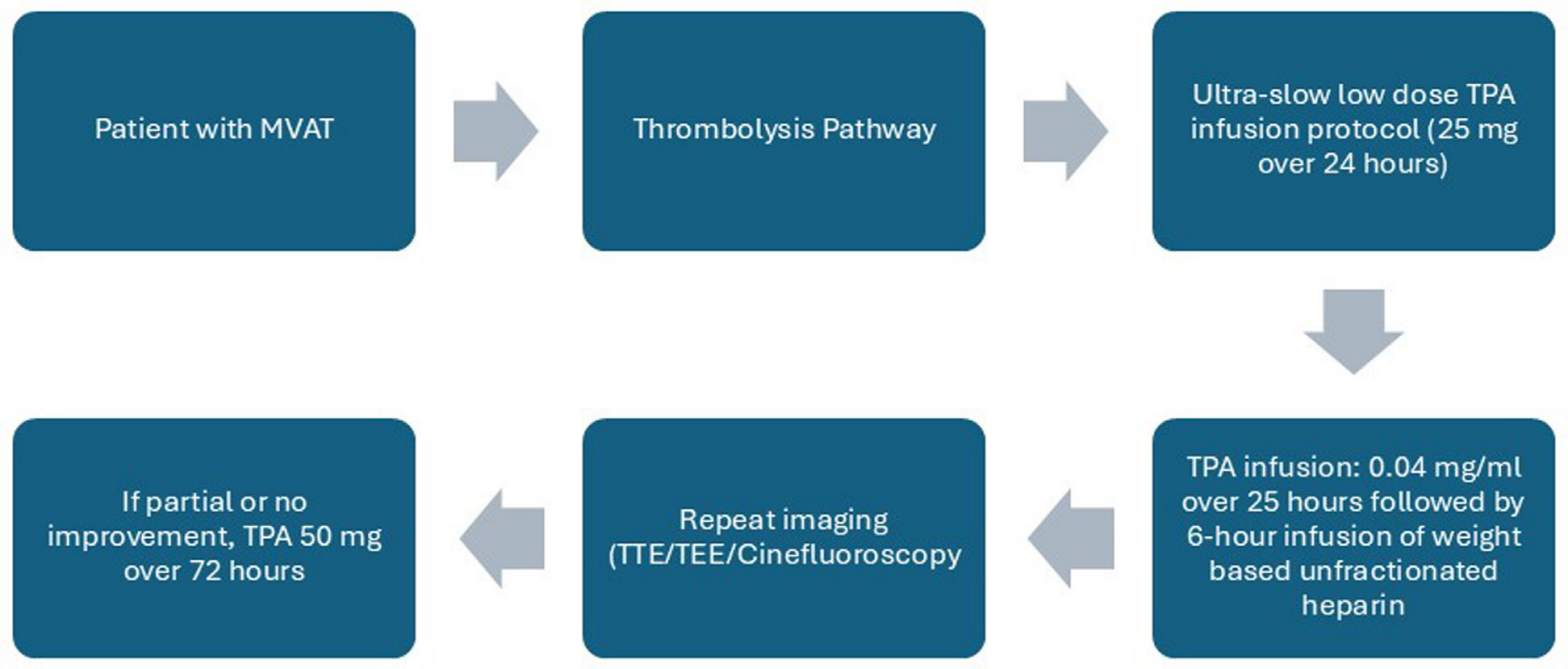
Algorithm for Management of Mechanical Aortic Valve Thrombosis Using Thrombolytics

Figure 5 summarizes the treatment protocol used. The patient was successfully transitioned to warfarin with an increased target INR of 2.5-3.5 based on the 2020 AHA/ACC guidelines.^2^ He remained hemodynamically stable and achieved complete resolution of symptoms, leading to his discharge on day 15 of admission after reaching the goal INR of 3.

## Discussion

MAVT is a rare but potentially life-threatening complication that can occur despite appropriate anticoagulation.^6^ In this case, our patient developed MAVT despite having an INR within the therapeutic range, highlighting that another factor, such as low cardiac output or transient subtherapeutic anticoagulation levels, may contribute to thrombus formation.

Traditionally, the management of MAVT has included both surgical and nonsurgical approaches.^2^ Surgical options, such as valve replacement or thrombectomy, are often considered the definitive treatment but carry significant risks with a 30-day mortality of 10% to 15%.^2^^,^^7^ Mechanical valve thrombosis treated with thrombolytics have shown a lower mortality than the surgical route (6.6% vs 18.1%, respectively).^8^

Thrombolytic therapy, using agents such as tPA, offers a nonsurgical alternative and the ACC/AHA Joint Committee on Clinical Practice Guidelines suggests fibrinolytic therapy as a Class 1 recommendation.^2^ The TROIA (TEE-Guided Thrombolytic Regimens for Prosthetic Valve Thrombosis) trial observed that low-dose (25 mg) slow infusion of tPA over 6 hours, repeated as needed, provided a safe and effective treatment option for patients with prosthetic valve thrombosis. ^9^ The PROMETEE trial used an ultra slow infusion over 25 hours of low-dose (25 mg) tPA and had a success rate of 90% (95% CI: 0.85-0.95).^5^ The multicenter HATTHUSHA (Thrombolysis or Surgery in Patients With Obstructive Mechanical Valve Thrombosis) study compared thrombotic therapy with either slow or ultra slow infusion of low-dose tPA and surgery for patients with obstructive mechanical valve thrombosis. Thrombotic therapy was associated with high success rates and lower complications and mortality rates.^10^

Another viable treatment option is a percutaneous transcatheter intervention for the patient population who have either failed thrombolysis or have a contraindication to both thrombolysis and a redo surgery. Available data and case reports have discussed percutaneous intervention mostly for a stuck mitral valve.^11^^,^^12^ In a single-center retrospective study, 24 patients with acute mechanical mitral valve thrombosis who failed thrombolysis, had contraindications for thrombolysis, or were high risk for surgery underwent the transcatheter release of a stuck mitral valve. Promising results were seen with procedural success (91.67%, n = 22), all-cause death (12.50%, n = 3), stroke (4.17%, n = 1), and recurrence (16.67%, n = 4).^13^ Another case series of 5 patients depicted good results with transcatheter balloon dilatation technique to release the stuck mitral valve.^14^

## Management Overview

• *Surgical approach:* Surgery remains the standard treatment, particularly for patients with large thrombi (>0.8 cm^2^), severe symptoms (NYHA functional class IV), or contraindications to thrombolysis. However, it is associated with high perioperative risks, especially in emergent cases.
• *Thrombolytic therapy:* Thrombolysis with agents such as recombinant tPA or streptokinase is increasingly used in patients who are not suitable for surgery. The choice of regimen and infusion rate significantly influences success rates and complications. Table 1 discusses the complications of TT and Table 2 discusses the current dosing regimens for TT.
  ○ *Fast regimens:* Thrombolytics delivered over hours provide rapid resolution but with a higher risk of embolic and hemorrhagic events.
  ○ *Ultra slow regimens:* Prolonged infusion at low doses offers better safety profiles and comparable efficacy.

**Table 1:** Complications of Thrombolytic Therapy

Complication	Details
Hemorrhagic complications	Intracranial hemorrhage, gastrointestinal bleeding, and retroperitoneal bleeding.
Hypotension	Related to systemic administration of thrombolytics.
Allergic reactions	Rare but include anaphylaxis, urticaria, and angioedema.
Reperfusion arrhythmias	Ventricular arrhythmias can occur during or after thrombolysis.
Systemic bleeding	Includes oozing from puncture sites, hematuria, and other systemic bleeding manifestations.
Resistance or reocclusion	Failure to achieve or sustain arterial patency after thrombolytic therapy.

Data from Kalidoss et al (2021).^15^

**Table 2:** Dosing Regimens of Thrombolytic Therapy

Regimen	Dosage	Duration	Key Features
Ultra slow regimen	25 mg rtPA	25 h	High efficacy (85% success), lowest complication rates, suitable for safer management.
Fast regimen	50 mg rtPA	6 h	Comparable efficacy (78.3% success), higher complication rate including intracranial hemorrhage.
Repeat doses	25 mg rtPA	6 h per cycle	Repeated up to a cumulative dose of 150 mg or until a complication occurs.

Data from Özkan et al (2013).^9^

rtPA = recombinant tissue plasminogen activator.

The decision to use thrombolytic therapy must balance the risk of bleeding against the potential benefits of thrombus resolution.^13^ Our patient was treated with a slow tPA infusion, starting at a rate of 25 mg over 25 hours (1 mg/hour). The initial partial improvement observed on echocardiography warranted a second tPA infusion. Ultra slow tPA infusion allowed for gradual thrombus dissolution, reducing the risk of the embolic complications that can occur with rapid thrombolysis. In our patient, the careful monitoring and controlled administration of tPA, followed by bridging anticoagulation therapy with heparin and warfarin, allowed for safe and effective management.

## Future Directions

Thrombolytic regimens need to be optimized, and long-term outcomes research also is needed that includes patients treated with thrombolytic therapy.

## Conclusions

MAVT is a rare complication requiring a high index of suspicion for timely diagnosis and management. This review highlights 2 key points. First, multimodality imaging in diagnosis is important, and a low-dose ultra slow infusion of tPA offers both efficacy and safety. Recent studies suggest that thrombolytic therapy offers comparable success rates with reduced mortality and bleeding risks in appropriately selected patients (Table 3). Our case demonstrates the efficacy of tPA as a treatment option for MAVT in a young patient with a complex cardiac history. The successful resolution of the thrombus and the patient’s subsequent stabilization highlight the potential of thrombolytic therapy to manage MAVT effectively, avoiding the immediate risks associated with repeat surgical intervention.

**Table 3:** Surgery Versus TT for Treatment of MAVT

First Author (Year)	Study Design	Study Group	No. of Studies/Patients	Success Rate	Death Rate	CNS ± Systemic Embolism	Major Bleeding	Recurrent MAVT
Karthikeyan et al (2013)^16^	Systematic review and meta-analysis	Urgent surgery	7 studies/n = 690 (surgery: 446; TT: 224)	86.5%	13.5%	1.6%	1.4%	7.1%
		TT		69.7%	9.0%	16%	5%	25.4%
Castilho et al (2014)^8^	Systematic review and meta-analysis	Surgery	48 studies/n = 2,239 (surgery: n = 1,132; TT: n = 1,107)	81.9%	18.1%	4.3%	4.6%	NA
		TT		80.7%	6.6%	5.6%	6.8%	NA
Özkan et al (2022)^10^	Observational prospective study (HATTUSHA)	Surgery	83	NA	18.7%	5.3%	9.3%	6.7%
		TT	75	90.4%	2.4%	2.4%	2.4%	2.4%

CNS = central nervous system; MAVT = mechanical aortic valve thrombosis; NA = not applicable; TT = thrombolytic therapy.

## Funding Support and Author Disclosures

The authors have reported that they have no relationships relevant to the contents of this paper to disclose.

## Appendix

Video 1 Left heart catheterization showed 100% occlusion of a dominant left circumflex artery in the distal atrioventricular groove before the posterior descending artery branch.

Video 2 A cinefluoroscopy was performed in the cardiac catheterization laboratory, showing an immobile anterior mechanical aortic leaflet.

Video 3 Cinefluoroscopy performed after the tissue plasminogen activator infusion revealed markedly improved valve opening.
